# Utility of the Venous Excess Ultrasound (VEXUS) score to track dynamic change in volume status in patients undergoing fluid removal during haemodialysis – the ACUVEX study

**DOI:** 10.1186/s13089-024-00370-9

**Published:** 2024-03-27

**Authors:** Adrian Wong, Olusegun Olusanya, Jim Watchorn, Kate Bramham, Sam Hutchings

**Affiliations:** 1 Department of Critical Care, King’s College Hospital, London, UK; 2 Department of Intensive Care Medicine, St. Bartholomew’s Hospital, London, UK; 3 Intensive Care Department, Royal Berkshire Hospital, Reading, UK; 4 Department of Women and Children’s Health and Centre for Urology, Nephrology and Transplantation, King’s College Hospital, London, UK

**Keywords:** Ultrasound, Venous congestion, Fluid overload, Renal replacement therapy

## Abstract

**Background:**

The use of ultrasound assessment, including the Venous Excess Ultrasound (VEXUS) score, is increasingly being utilised as part of fluid status assessment in clinical practice. We aimed to evaluate the ability of the VEXUS score to track fluid removal during the course of the dialysis session and explore the relationship between traditional measures of fluid status and venous congestion.

**Methods:**

Single-centre, observational study in patients undergoing intermittent haemodialysis, who presented above their target dry weight. Patients had serial assessment using VEXUS, lung ultrasound and selected echocardiographic measures, before, during and after fluid removal.

**Results:**

Amongst 33 patients analysed, 5 (15%) had an elevated VEXUS score (> 0). There was no difference in starting weight, dry weight or amount of fluid removed in patients with a normal VEXUS score and those with an elevated VEXUS score. In all patients with elevated VEXUS scores, the degree of venous congestion improved during the course of fluid removal. All patients with an elevated VEXUS score had evidence of both right and left ventricular systolic impairment.

**Conclusion:**

In patients with ESRF undergoing haemodialysis, the incidence of venous congestion as measured by the VEXUS is low. In patients with elevated VEXUS scores, removal of fluid through haemodialysis improves the venous congestion score. The pattern of LV and RV systolic dysfunction suggests that VEXUS may be a reflection of cardiac failure rather than venous volume status.

**Trial registration:**

Ethical approval was provided by South Central-Berkshire Research and Ethics Committee and registered on clinicaltrials.org (IRAS305720). Trial registration: ISRCTN14351189 – Retrospectively registered on 30/11/2023.

## Introduction

Fluids are the most commonly administered intravenous therapy in patients on the intensive care unit (ICU); indeed, management of fluid status is a fundamental aspect of critical care. Whilst research mainly focussing on the administration of fluid during the resuscitation and optimisation phase continues to expand, management strategies to detect fluid overload and guide fluid removal remain sparse [1]. A positive fluid balance in critically ill patients is associated with poorer outcomes in a variety of conditions [2–4].

Available physiological parameters and monitoring devices have not evolved beyond weighing patients, documenting cumulative fluid balance and clinical examination of oedema [5]. Hence, there is significant variation in practice amongst clinicians with regards to the physiological parameters to monitor fluid status and subsequently guide management [6].

The role of venous congestion in various organ dysfunction is gaining prominence although the degree to which it contributes to the pathophysiology is unclear. Part of this knowledge gap can be attributed to the lack of validated techniques to accurately diagnose and monitor venous congestion, in order to study the condition [7].

Recently, Beaubien-Souligny and colleagues developed the Venous Excess Ultrasound Score (VEXUS) which involves a structured ultrasound evaluation of venous congestion [8]. The scoring system has been shown to predict the incidence of acute kidney injury in the post-cardiac surgery patient population. Despite increasing interest, the incidence of high VEXUS score in the general ICU population is low [9] and few studies have explored the validity of the score in a wider setting [10]. This issue is further compounded by the interchangeable use of the various terms used to describe fluid excess e.g. fluid overload, congestion, oedema etc. [11].

Patients undergoing intermittent haemodialysis are in established kidney failure (ESRF). This group of patients is more homogenous compared to patients within intensive care who are undergoing renal replacement therapy. Furthermore, these patients are more likely to be fluid overloaded (index condition) at the outset of their scheduled dialysis session. Optimising fluid balance in such patients, though challenging, is potentially beneficial as it reduces myocardial stretch and remodelling, improve cognition, reduce fatigue etc. [12]. During the treatment period, the majority of these patients will have fluid removed through the extra-corporeal circuit; this would permit assessment of the ultrasonographic appearances in response to this procedure [13].

The primary aim of the study was to evaluate the ability of the VEXUS score to track fluid removal during the course of the dialysis session and explore the relationship between patient’s weight pre-dialysis and venous congestion. The secondary objective was to examine the influence of right and left ventricular parameters on the score.

## Methodology

The study was conducted in the renal dialysis unit of a tertiary-level hospital in London, United Kingdom between May and October 2022. Adult patients with end-stage renal failure, on maintenance haemodialysis, who presented above their target dry weight and had a target fluid removal of 2 L, or more, were included. Exclusion criteria were patients with previous echocardiographic evidence of right heart dysfunction, previous liver resection or liver transplantation, known liver cirrhosis and pregnancy. The trial was conducted according to principles set out by the Helsinki Declaration and ethical approval was provided by South Central-Berkshire Research and Ethics Committee (IRAS305720); written informed consent was obtained from each patient.

### Ultrasound assessment

All ultrasound examinations were performed using an Affiniti Ultrasound System (Philips, UK). All examinations were performed by clinicians accredited to a minimum of UK focused critical care competencies who had been additionally trained in VEXUS through lectures and videos. They were not part of the treating team. The examinations were performed before the start of fluid removal, during, and at the end of their dialysis session.

Cardiac ultrasound examinations were performed using a standard phased-array probe (2–5 MHz) to obtain the standard parasternal long-axis, short-axis, apical and subcostal windows. The left ventricular outflow tract velocity time integral (LVOT VTi) was obtained by placing the pulse-wave Doppler gate at the left ventricular outflow tract in the apical 5-chamber view. We defined LV systolic impairment as LVOT VTi < 16 cm. The RV systolic function was defined according to the tricuspid annular plane systolic excursion (TAPSE) assessed using M-mode recordings through the lateral tricuspid valve annulus. In addition, tissue doppler analysis was performed to measure myocardial velocity change using standard pulse-wave Doppler at the junction of the RV free wall and tricuspid annulus in the apical 4-chamber view.

Lung ultrasound examinations were performed using a curvilinear probe (1-5Mhz) using a previously described protocol [14]. The probe was placed in the longitudinal plane across the rib space in 8 segments. The lung ultrasound score was obtained, with each segment scored between 0 and 3 based on the primarily the number of B-lines. We defined a ‘Wet’ profile if there were bilateral segments of lungs which scored 2 or more.

**Fig. 1:**
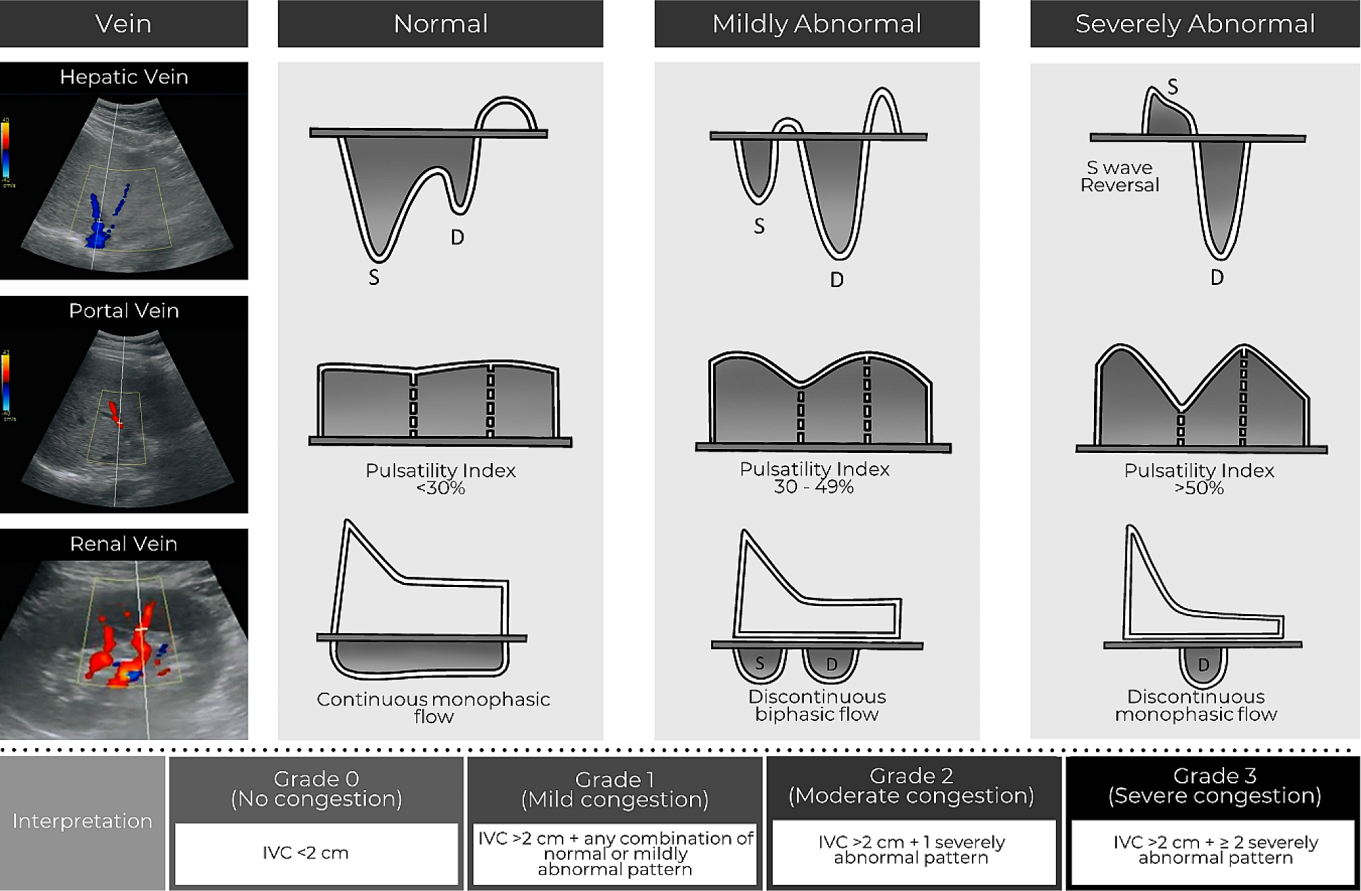
Grading of HV, PV and intrarenal vein Doppler patterns. VEXUS Ultrasound Score: Grade 0: IVC < 2 cm = NO Congestion; Grade 1: IVC > 2 cm with any combo of Normal or Mildly Abnormal Patterns; Grade 2: IVC > 2 cm and ONE severely Abnormal Pattern; Grade 3: IVC > 2 cm and > 2 Severely Abnormal Patterns

Assessment of venous congestion was performed using Doppler-based techniques as previously described by Denault et al. [15] (Fig. 1). Briefly, with the patient in the supine position, the diameter of the inferior vena cava was measured in the subcostal view at 1 cm from its junction with the right atrium. The maximum and minimum diameters of the inferior vena cava were measured, and the percentage of change in diameter was calculated. The hepatic venous (HV) flow was recorded from the subcostal window. HV patterns were classified and recorded as: continuous, systolic greater than diastolic (S > D, normal), systolic less than diastolic (S < D, abnormal) or systolic reversal (severely abnormal).

Portal vein pulsatility was assessed by pulsed-wave Doppler evaluation of the portal vein (PV) in the liver either in the subcostal or lateral position. PV patterns include continuous (normal), or pulsatile. Furthermore, where possible, the pulsatility index (PI) was calculated: ([PVmax – Pvmin]/PVmax × 100%).

The measurements were categorised based on the Doppler appearance and used to calculate the VEXUS grade (0–3).

A modification of the VEXUS grade in our study was the omission of the intrarenal flow profile. We had intended to include this parameter but after recruiting several patients it became apparent that renal vein flow was mostly unrecordable. The rationale for this is the presumed alterations of renal perfusion in the context of ESRF and hence its unknown impact on the validity/feasibility of the technique [15–17].

Images and videos were de-identified with respect to patient, and time point and were analysed offline in a blinded fashion by clinicians who did not perform the scans.

### Additional data collection

Basic demographic data were collected from the patients’ records. The target dry weight had been previously decided by the patient’s treating nephrologist. Before the dialysis session, patients are weighed, and fluid removal target decided, so as to achieve their target dry weight. Basic parameters such as blood pressure and heart rate were recorded.

### Statistical analysis

Statistical analysis was conducted using Prism v 10.1 (GraphPad Software, San Diego, CA).

Data on dynamic change in VEXUS parameters is not available, precluding a formal power calculation. We therefore pragmatically aimed to recruit 30 patients. Continuous variables are expressed as the mean ± standard deviation or the median and interquartile range, depending on distribution of the data. Categorical variables are presented as frequencies and proportions. Differences between groups at baseline were analyzed using the t-test or Wilcoxon-Mann‒Whitney U test according to normality criteria. Fisher’s exact test was applied to categorical variables.

## Results

60 consecutive patients were screened. The main reasons for exclusion were documented right ventricular dysfunction and co-existing liver disease. 45 patients were eligible, and 35 patients were recruited (10 patients refused consent); two patients were excluded from final analysis due to poor ultrasound windows. 33 patients were included for final analysis.

### Demographics (Table 1)

The mean age of the study group was 63. There were 20 males and 13 females in the final analysis. The most common aetiology for the ESRF was hypertension.

The mean delta weight (starting – dry weight) and target fluid removal were 2.9 kg and 2.4 L respectively.

At baseline, there were 7 patients with left ventricular systolic impairment (LVOT VTi < 16 cm) and 6 patients with right ventricular systolic impairment (TAPSE < 1.6 cm or RV S’ < 10 cm/s).

**Table 1:** Baseline characteristics of cohort. Mean ± Standard Deviation (SD). Median (Interquartile range)

	VEXUS 0 (*n* = 28)	VEXUS > 1 (*n* = 5)	p-value
Age (years)	64 ± 11	60 (15)	0.61
Women, n (%)	12 (42.9)	1 (20)	0.62
**Aetiology of end-stage renal failure**			
Hypertension	17	3	
Diabetes mellitus	12	0	
Focal segmental glomeruloronephritis	5	0	
IgA nephropathy	2	0	
Adult polycystic kidney disease	1	1	
Tubulointestialnephritis	0	1	
Uncertain	1	0	
**Type of dialysis access**			
Arteriovenous fistula	19	3	0.99
Tunnelled line	9	2	
Dry weight (kg), mean (SD)	77 (14)	88 (22)	0.17
Starting weight (kg), mean (SD)	80 (14)	90 (21)	0.17
Delta weight (Starting weight – dry weight, kg), mean (SD)	2.95 (2.9)	2.78 (1.35)	0.89
Heart rate (bpm), mean (SD)	70 (11)	74 (15)	0.44
Systolic blood pressure (mmHg), median (IQR)	144 (60.7)	134 (31)	0.54
Diastolic blood pressure (mmHg), median (IQR)	75 (30.5)	72 (27.5)	0.53

### Ultrasound assessment

#### VEXUS grade

5 patients (15%) had a VEXUS grade of > 1 at the start of the dialysis session. These were all VEXUS grade 3. 2 further patients had abnormal HV and PV Doppler flow patterns but an IVC < 2 cm, leading them to be categorised as having a VEXUS score of 0.

When individual components (IVC, HV and PV) of the VEXUS score were analysed, the incidence of abnormal measurements were 15% (*n* = 5), 21% (*n* = 7) and 21% (*n* = 7) respectively.

There was no difference in either dry weight, delta weight or targeted fluid removal between patients who had VEXUS grade 0 and those > 1. Patients with VEXUS grade > 1 had more fluid removed compared to those with VEXUS grade 0.

**Fig. 2:**
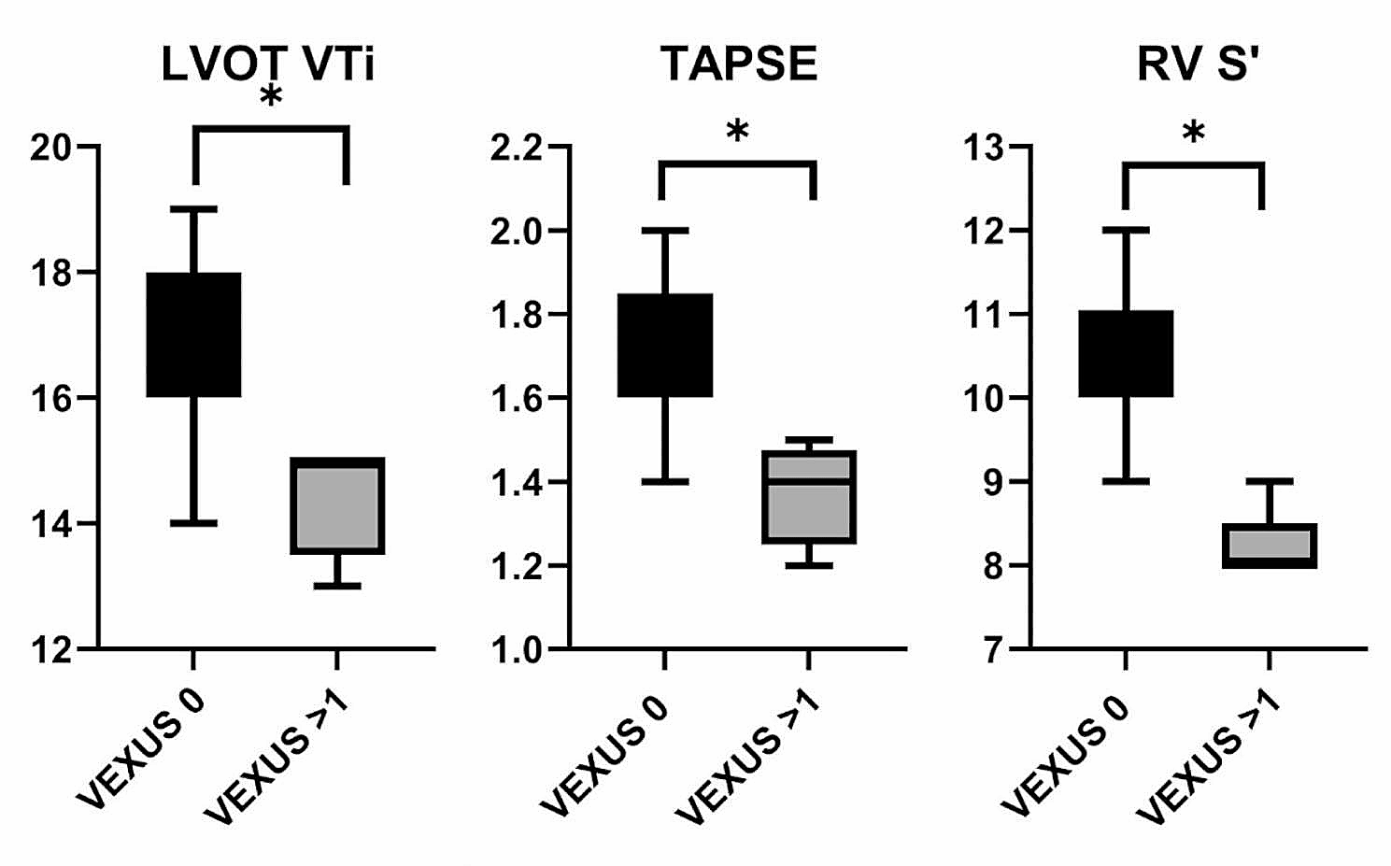
Baseline differences in LV and RV parameters in VEXUS 0 and VEXUS > 1 groups. * *p* < 0.05

There was a significant difference in the incidence of left and right ventricular systolic impairment between the two groups. All patients with VEXUS > 1 had impaired RV systolic function (Table 2; Fig. 2).

**Table 2:** Ultrasound parameters at baseline

Ultrasound findings	VEXUS 0	VEXUS > 1	p-value
**LV systolic function**			
Normal (n)	26	0	0.0001
Abnormal (n)	2	5	
LVOT VTi (cm), mean	17.1 (1.3)	14.4 (0.9)	< 0.0001
RV S’ (cm/s), mean (SD)	10.7 (0.8)	8.2 (0.4)	< 0.0001
TAPSE (cm), mean (SD)	1.7 (0.1)	1.4 (0.1)	0.0002
**RV systolic function**			
Normal (n)	27	0	0.0001
Abnormal (n)	1	5	
**Lung ultrasound**			
Normal (n) (%)	27 (%)	3	0.0001
Abnormal (n)	1	2	
**VEXUS Grade (n)**			
0	28	0	
1	0	0	
2	0	0	
3	0	5	

There were 3 patients (2 in the VEXUS 0 group and 1 in the VEXUS > 1 group) who had ultrasonographic evidence of pulmonary congestion.

#### Dynamic change in VEXUS score

In the 5 patient who had VEXUS grade > 1, all 5 had an improvement in VEXUS grade at the end of dialysis session.

In all patients who had any ultrasonographic evidence of congestion (HV, PV and lung), the degree of abnormality improved with fluid removal.

**Fig. 3:**
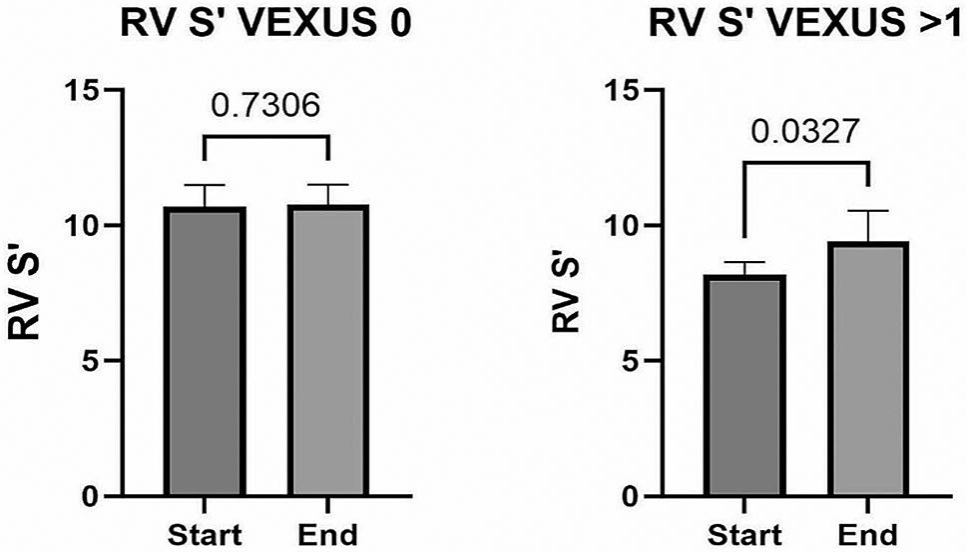
LV and RV parameters at the start and end of haemodialysis session in VEXUS 0 and VEXUS > 1 group

With regards to LV and RV parameters, there was no significant difference at the start and end of fluid removal except in RV S’ in the VEXUS > 1 group (Fig. 3).

## Discussion

In the present study, we report on the use of the VEXUS point of care ultrasound tool to both assess intravascular volume status against traditional parameters of volume status, such as weight and its utility in guiding volume removal.

Although limited by the small size of the group with elevated VEXUS scores prior to fluid removal, it was interesting to observe that patients with an elevated VEXUS score did not appear to also manifest features traditionally used to mark patients as volume overloaded such as the delta between dry and actual weight. If Intra vascular or intra venous volume was in equilibrium with total body volume status, then one might expect there to be a relationship between these factors that was not evident in the current study. The finding that patients with high VEXUS scores were also more likely to have ultrasonographic evidence of pulmonary oedema may suggest that these objective tools are more helpful in clinically delineating significant volume overload than more traditional parameters.

In the current study, all patients with ultrasonographic evidence of congestion demonstrated ultrasonographic improvements during the haemodialysis session. This was true for both VEXUS and lung ultrasound scores. The use of ultrasound, especially lung ultrasound, as an objective, repeatable assessment of the efficacy of fluid removal in HD patients is gaining popularity [18, 19]. Alexiadis et al. demonstrated the utility of lung ultrasound over other methods for evaluating dry weight and fluid status and helping recognize asymptomatic lung congestion (AUROC 0.81–0.83) [20]. In the present study, 2 patients had a VEXUS grade of 0 but demonstrated ultrasonographic evidence of pulmonary congestion. In these 2 patients, again the ultrasonographic appearance improved with fluid removal. There is evidence to suggest that such subclinical pulmonary oedema is associated with poorer outcomes in patients with heart failure [21].

The use of VEXUS scores to track/monitor fluid removal using diuretics and renal replacement therapy has only been described in small case series [22]. There are no observational or randomised-controlled trials evaluating VEXUS performance. The AKIVEX study [23] concluded that addition of the VEXUS score in the management of critically ill patients with severe AKI allowed the identification of patients with venous congestion and provided greater diuretic use in these patients, resulting in significantly more RRT-free days in 28 days, in patients who reduced the VEXUS score. The authors concluded that VEXUS can be used as an indicator for, and monitoring during fluid removal. However, the study was limited by the fact that it was unblinded, there was no standardised approached to treatment post scan and the relationship between VEXUS and cardiac function was not evaluated. The results of the present study provides evidence that VEXUS and lung ultrasound may be of clinical use in objectively tracking fluid removal.

A key finding of the present study is that all 5 patients who had an elevated VEXUS score at inclusion had evidence of both RV and LV systolic dysfunction. Despite the obvious limitation of the small numbers in this group it raises the possibility that rather than being a measure of volume status, abnormal VEXUS score may instead be a reflection of ventricular dysfunction. Longino and colleagues showed a correlation between VEXUS grade and right atrial pressures in their pilot observational study of patients undergoing cardiac surgery [24, 25], but did not assess if interventions affecting one, is reflected in the other.

Interestingly, improvement in venous congestion, as evidenced by VEXUS score, was not reflected in improvement in LVOT VTi and TAPSE following fluid removal. It therefore raises the question of whether VEXUS grade improvement precedes improvement in RV and LV systolic parameters. An alternative explanation is that the improvements in the VEXUS score may not be due to the improvements in RV/LV performance from fluid removal. To our knowledge, no other study has evaluated the association and temporal relationship between VEXUS and cardiac function.

There are a number of limitations to the present study. The single centre design and clinical stability of the patients may limit the wider generalisability of the results. Crucially, despite presenting above their dry/target weight and having at least 2 L of fluid removed during the haemodialysis session, the incidence of an elevated VEXUS score, at baseline, was low and this limits the ability to draw firm conclusions from the data. We did not include the assessment of intrarenal venous Doppler, although this formed part of the initially described VEXUS score. There are several reasons for this; previous studies have shown that intrarenal venous Doppler was limited in the context of CKD (Wiersema and Spiegel), it is unknown whether patients with ESRF on IHD have alterations in renal blood flow and alterations in PV correlate with intrarenal venous Doppler data [15–17].

## Conclusion

In patients with ESRF undergoing haemodialysis and fluid removal, the incidence of venous congestion as measured by the VEXUS is low. In patients with elevated VEXUS scores, removal of fluid through haemodialysis improves the venous congestion score.

The use of weight to guide fluid status and hence removal does not correspond to the ultrasonographic appearance of venous congestion as measured by the VEXUS score. The pattern of LV and RV systolic dysfunction suggests that VEXUS may be a reflection of cardiac failure rather than fluid status per se.

The VEXUS score is primarily a quantitative analysis of the IVC, HV, PV and intrarenal vein. The impact of qualitative analysis of Doppler flow as well as the relative contribution/importance of each component requires further studies.

The impact of incorporating assessment of venous congestion (such as venous ultrasound) to guide fluid management in both the critically ill and in those undergoing haemodialysis is still unknown and should be evaluated in future studies.

## Data Availability

The datasets used and/or analysed during the current study are available from the corresponding author on reasonable request.

## Abbreviations

AKI: Acute Kidney Injury
ESRF: End stage renal failure
HV: Hepatic vein
ICU: Intensive Care Unit
IVC: Inferior vena cava
LV: Left ventricle
PV: Portal vein
RV: Right ventricle
TAPSE: Tricuspid annular plane systolic excursion
VEXUS: Venous Excess Ultrasound
VTi: Velocity time integral
